# Butyrate Transcriptionally Enhances Peptide Transporter PepT1 Expression and Activity

**DOI:** 10.1371/journal.pone.0002476

**Published:** 2008-06-25

**Authors:** Guillaume Dalmasso, Hang Thi Thu Nguyen, Yutao Yan, Laetitia Charrier-Hisamuddin, Shanthi V. Sitaraman, Didier Merlin

**Affiliations:** Department of Medicine, Division of Digestive Diseases, Emory University School of Medicine, Atlanta, Georgia, United States of America; University of Hong Kong, China

## Abstract

**Background:**

PepT1, an intestinal epithelial apical di/tripeptide transporter, is normally expressed in the small intestine and induced in colon during chronic inflammation. This study aimed at investigating PepT1 regulation by butyrate, a short-chain fatty acid produced by commensal bacteria and accumulated inside inflamed colonocyte.

**Results:**

We found that butyrate treatment of human intestinal epithelial Caco2-BBE cells increased human PepT1 (hPepT1) promoter activity in a dose- and time-dependent manner, with maximal activity observed in cells treated with 5 mM butyrate for 24 h. Under this condition, hPepT1 promoter activity, mRNA and protein expression levels were increased as assessed by luciferase assay, real-time RT-PCR and Western blot, respectively. hPepT1 transport activity was accordingly increased by ∼2.5-fold. Butyrate did not alter hPepT1 mRNA half-life indicating that butyrate acts at the transcriptional level. Molecular analyses revealed that Cdx2 is the most important transcription factor for butyrate-induced increase of hPepT1 expression and activity in Caco2-BBE cells. Butyrate-activated Cdx2 binding to hPepT1 promoter was confirmed by gel shift and chromatin immunoprecipitation. Moreover, Caco2-BBE cells overexpressing Cdx2 exhibited greater hPepT1 expression level than wild-type cells. Finally, treatment of mice with 5 mM butyrate added to drinking water for 24 h increased colonic PepT1 mRNA and protein expression levels, as well as enhanced PepT1 transport activity in colonic apical membranes vesicles.

**Conclusions:**

Collectively, our results demonstrate that butyrate increases PepT1 expression and activity in colonic epithelial cells, which provides a new understanding of PepT1 regulation during chronic inflammation.

## Introduction

Butyrate is a short-chain fatty acid (SCFA) produced in the colonic lumen by bacterial fermentation of carbohydrates and dietary fibers [1]. In normal mammals, the colonic lumen contains 100–150 mM total SCFAs [2], [3]. The molar ratio of three major SCFAs acetate, propionate and butyrate (which constitute approximately 90% of total SCFAs in the lumen varies due to several factors but is generally about 60∶20∶20 for acetate∶propionate∶butyrate [3], [4]. In mammals, 95–99% of SCFAs produced in the colonic lumen are absorbed [4], [5]. The pH of colonic lumen is about 6.2, suggesting that at least 90% of all SCFAs exist under ionized forms. SCFAs uptake by epithelial cells occurs by simple diffusion of the unionized forms across cell membranes, whereas uptake of the ionized form is mediated by apical membrane monocarboxylic transporter (MCT)-1 [6]. SCFAs are rapidly metabolized by colonocytes and are the main respiratory fuels in the intestine; indeed, oxidation of SCFAs supplies 60–70% of the energy need in isolated colonocytes [7]. Of the three major SCFAs, butyrate is the main intestinal fuel even in the presence of competing substrates such as glucose and glutamine [8]. In addition to its function as the dominant energy source for colonocytes, butyrate also affects cellular proliferation, differentiation and apoptosis [9], [10], [11].

Intestinal epithelial cells absorb small dietary peptides by the action of apical membrane peptide transporters. A cDNA encoding an apical membrane protein possessing this peptide transport capability has been cloned from humans and designated hPepT1 [12], [13], [14]. PepT1 is primarily expressed in brush border membranes of enterocytes in the small intestine, in the proximal tubular cells of the S1 segment of the kidney, and in bile duct epithelial cells [15], [16], [17]. Within the small intestine, PepT1 has a differential pattern of expression. Along the vertical axis, PepT1 is most abundant at the villous tip and expression decreases towards the crypt [18]. Along the longitudinal axis, the density of PepT1 increases from duodenum to ileum [19]. PepT1 is also expressed in immune cells as recently reported [20], [21]. PepT1 is generally not expressed in the esophagus, stomach or normal colon [16], [22]; however, hPepT1 expression has been observed in inflamed colon from patients with inflammatory bowel disease (IBD) [22] suggesting a link between hPepT1 expression and inflammatory pathways. Furthermore, hPepT1 expression was found to be upregulated in the colonic mucosa of patients with short-bowel syndrome after surgical resection of the proximal small intestine, which also indicates that hPepT1 expression is induced in colonic epithelial cells under pathological conditions [23]. In epithelial cells, butyrate is a potent stimulator of the transcription of membrane transporters such as the rat Na^+^/H^+^ exchanger (NHE3) [24], [25] and the human γ-epithelial sodium channel [26].

In the present study, we investigated the possibility that butyrate could induce colonic PepT1 expression and the underlying molecular mechanisms.

## Results

### Butyrate is a potent enhancer of hPepT1 promoter activity

**Figure 1:**
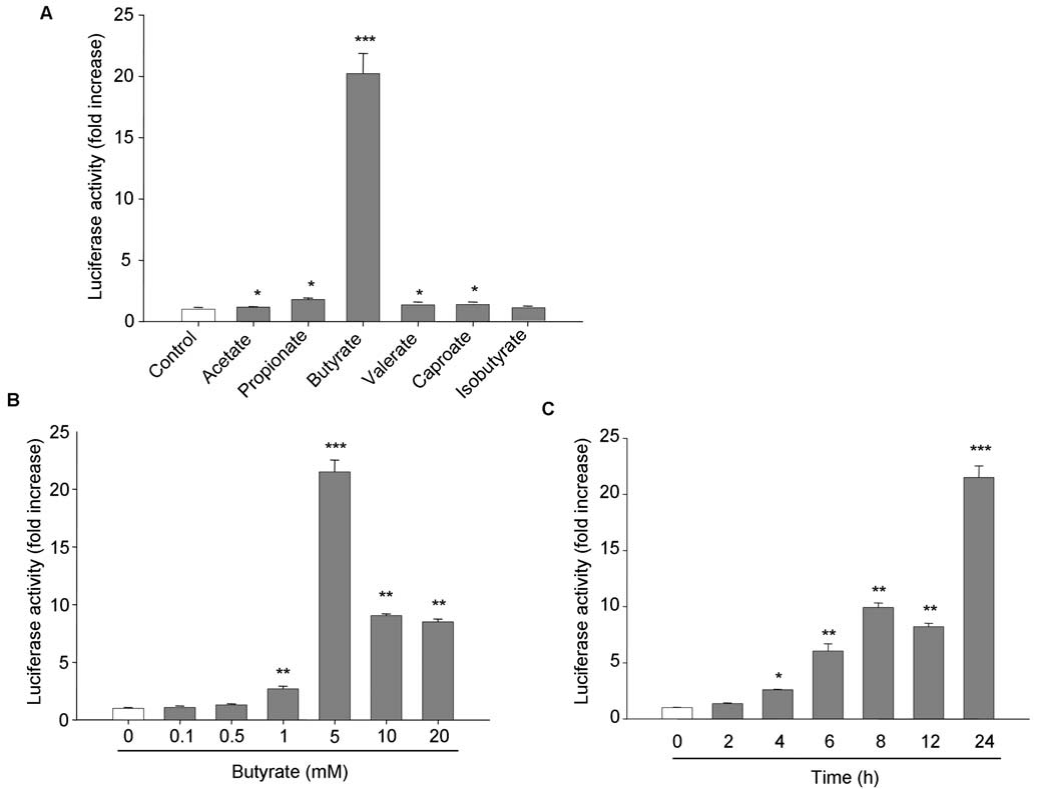
Butyrate increases hPepT1 promoter activity in Caco2-BBE cells. Caco2-BBE cells were transfected with full-length hPepT1 promoter construct and treated with A) 5 mM of the indicated short chain fatty acids for 24 h, B) the indicated concentrations of butyrate for 24 h, or C) 5 mM of butyrate for the indicated times. Luciferase activity related to hPepT1 promoter activity was measured. Data were normalized by Renilla activity and expressed as fold increases compared with untreated cells (control). Values represent means±S.E. of three determinations. *P<0.05; **P<0.005; ***P<0.001 *vs* control.

To test the response of the hPepT1 promoter to SCFAs, human intestinal epithelial Caco2-BBE cells were transfected with a reporter construct encoding the putative proximal promoter region of hPepT1 gene (722 bp) previously cloned in our laboratory ([27]; GeneBank™ accession number DQ370174). The transfected cells were treated or not with 5 mM of each SCFA: acetate, propionate, butyrate, valerate, capronate or isobutyrate, for 24 h. Luciferase activity relative to hPepT1 promoter activity was then assessed. Our results revealed that of the six examined SCFAs, butyrate was the most potent enhancer of hPepT1 promoter activity (Figure 1A). Furthermore, butyrate increased hPepT1 promoter activity in a dose- and time-dependent manner, with a maximal increase of ∼21-fold observed after 24 h exposure of cells with 5 mM butyrate (Figure 1B and C). Treatment with 5 mM butyrate for 24 h was therefore used for subsequent *in vitro* studies.

### Butyrate increases hPepT1 mRNA and protein expression

**Figure 2:**
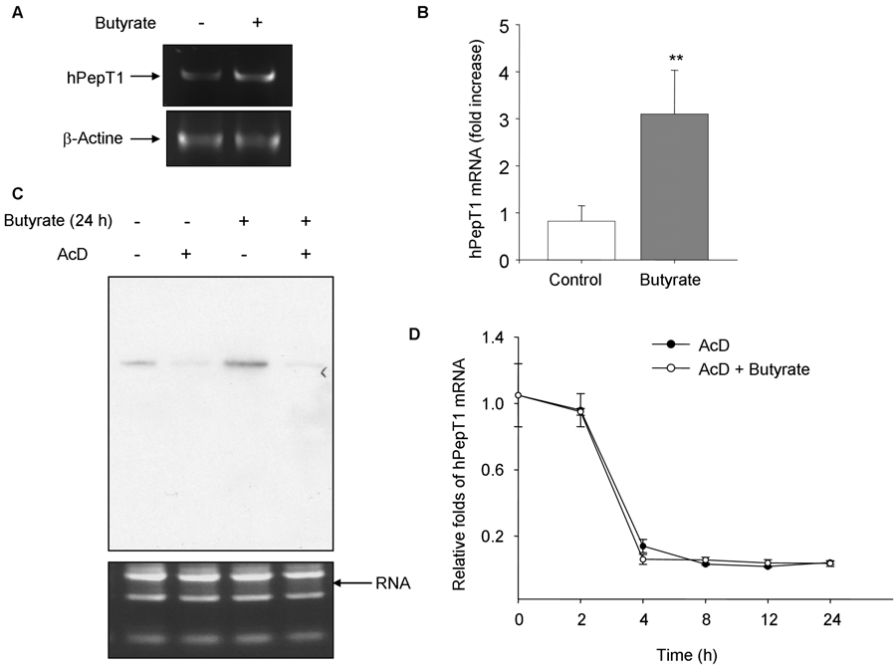
Butyrate transcriptionally up-regulates hPepT1 expression in Caco2-BBE cells. Caco2-BBE cells were treated with 5 mM butyrate for 24 h and hPepT1 mRNA levels were assessed by A) semi-quantitative RT-PCR and B) real-time RT-PCR. Values represent means±S.E. of three determinations. **P<0.005 *vs* control. To examine butyrate effect on the stability of hPepT1 mRNA, cells were pre-incubated with 5 µg/ml Actinomycin D (AcD) for 30 min and then treated with butyrate for 24 h. C) Total RNA was analyzed by Northern blot using a probe specific to the hPepT1 transcript. RNA loading controls were shown as bottom panel. D) hPepT1 mRNA levels were quantified using real-time RT-PCR. Values expressed as normalized cycling threshold values relative to untreated (0 h) cells represent means±S.E. of three determinations.

We then investigated the effect of butyrate on hPepT1 mRNA and protein expression levels in Caco2-BBE cells. As examined by RT-PCR, treatment of cells with 5 mM butyrate for 24 h strongly increased hPepT1 mRNA level (Figure 2A). In support of this result, real-time RT-PCR data showed that butyrate induces a significant increase of hPepT1 mRNA level by ∼3-fold compared to control cells (Figure 2B). We then examined whether the increase of hPepT1 mRNA level in butyrate-treated cells is due to changes in hPepT1 mRNA stability. hPepT1 mRNA expression levels in Caco2-BBE monolayers treated or not with 5 mM butyrate for 24 h in the presence or absence of 5 µg/ml actinomycin D (AcD), a potent transcription inhibitor, were analyzed by Northern blot. As shown in Figure 2C, the ∼2.2-kb band representing the hPepT1 mRNA was significantly increased in butyrate-treated cells compared with untreated cells (lane 3 *vs* lane 1). However, in the presence of AcD, hPepT1 mRNA was strongly reduced (lane 2) and the butyrate-induced increase of hPepT1 mRNA was suppressed (lane 4 *vs* lane 3). In agreement with this result, we found by real-time RT-PCR that hPepT1 mRNA levels in AcD-treated cells and AcD+ butyrate-treated cells were the same (Figure 2D). Together, these results showed that butyrate did not affect hPepT1 mRNA stability, demonstrating that it up-regulates hPepT1 at the transcriptional level.

**Figure 3:**
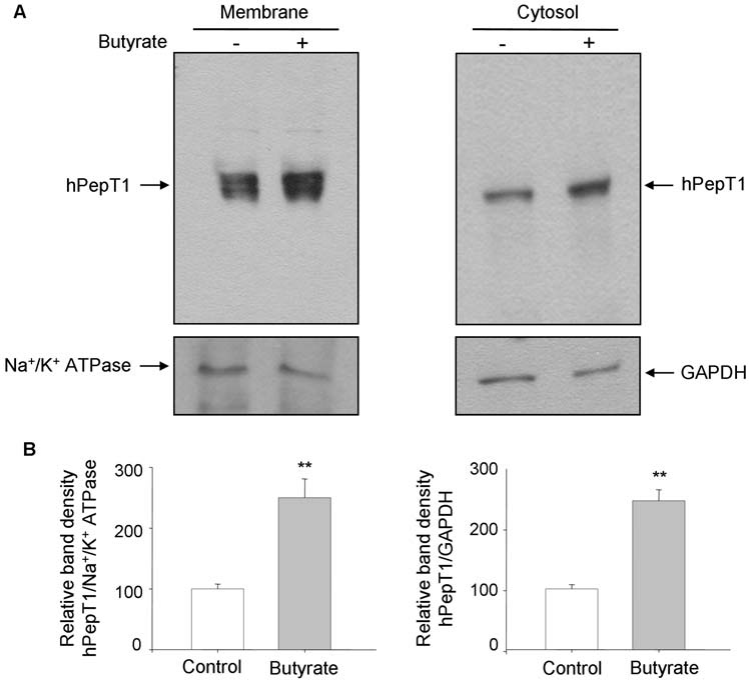
Butyrate increases hPepT1 protein expression in Caco2-BBE monolayers. A) Caco2-BBE cells grown on filters were treated with 5 mM butyrate for 24 h and membrane and cytosol hPepT1 protein expression was analyzed by Western blot. Expressions of Na^+^/K^+^ ATPase and GAPDH were used as loading controls. B) Bar graphs represent the densitometric quantification of hPepT1 blots shown in (A). Values represent means±S.E. of four blots from independent experiments. **P<0.005.

Furthermore, butyrate-treated Caco2-BBE monolayers exhibited greater hPepT1 protein expression level than untreated cells as examined by Western blot (Figure 3A). Densitometric analysis of hPepT1 band intensity revealed that both membrane and cytosol hPepT1 amounts were significantly increased by ∼2.5 fold upon butyrate treatment (Figure 3B).

Collectively, these data showed that butyrate transcriptionally up-regulates hPepT1 mRNA expression, resulting in the increase of hPepT1 protein level.

### Butyrate increases hPepT1-mediated dipeptide uptake in Caco2-BBE monolayers

**Figure 4:**
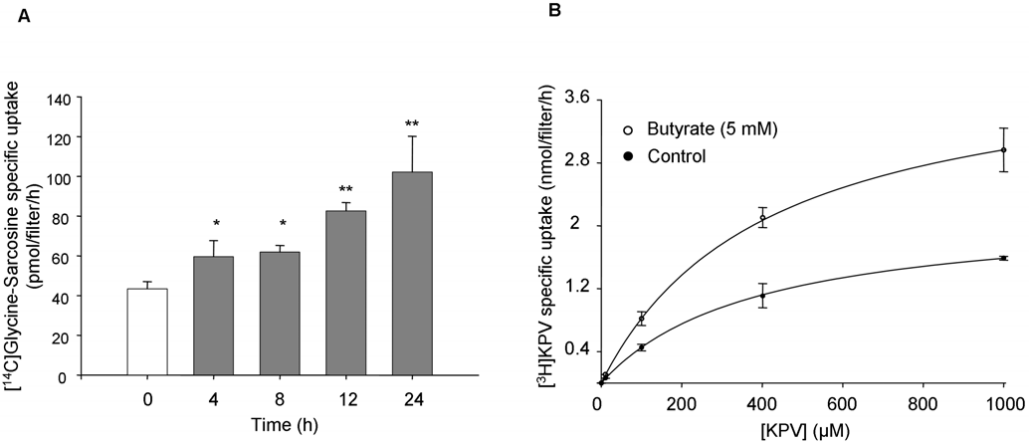
Butyrate increases hPepT1-mediated peptides uptake in Caco2-BBE monolayers. Caco2-BBE cells were grown on filters and apically treated with 5 mM butyrate for the indicated times. Uptake experiments were performed with 20 µM [^14^C]Glycine-Sarcosine±20 mM Glycine-Leucine at an apical pH of 6.2 and basolateral pH of 7.4 for 15 min at room temperature, and radioactivity was then measured. Values, expressed as fold increases compared with untreated cells, represent means±S.E. of three determinations. *P<0.05; **P<0.005 *vs* untreated cells. B) Caco2-BBE cells were grown on filters and apically treated with (○) or without (•) 5 mM butyrate for 24 h. Uptake experiments were performed using 20 nM, 120 nM, 10 µM, 100 µµ, 400 µµ and 1 mM of [^3^H]KPV. Results represent means±S.E. of three determinations.

Since butyrate induces an increase of hPepT1 expression, we next examined the effect of butyrate on hPepT1 transport activity. hPepT1-mediated Glycine-Sarcosine uptake was measured in Caco2-BBE monolayers pre-treated with 5 mM of butyrate for 4, 8, 12 and 24 h. As shown in Figure 4A, addition of butyrate to the apical compartment significantly increased hPepT1-mediated Glycine-Sarcosine uptake in a time-dependent manner. The increase of hPepT1 activity reached a maximal level of ∼2.5 fold compare to the basal level after 24 h treatment. To examine whether butyrate might affect the peptide transport by modifying the intrinsic activity of hPepT1, the kinetics of peptide transport in Caco2-BBE cells were examined in the presence or absence of butyrate (Figure 4B). The tripeptide KPV (Lys-Pro-Val) was used for its unique high affinity to PepT1 as previously demonstrated [21]. Kinetic analysis of the data indicated that butyrate significantly increased the maximal velocity V_max_ (4.2 nmol/filter/h vs 2.2 nmol/filter/h; P<0.05), but did not modify the Michaelis-Menten constant Km (408 µM vs 383 µM; non significant, P>0.05). These data exclude changes in the affinity of hPepT1 for peptides upon butyrate treatment.

**Figure 5:**
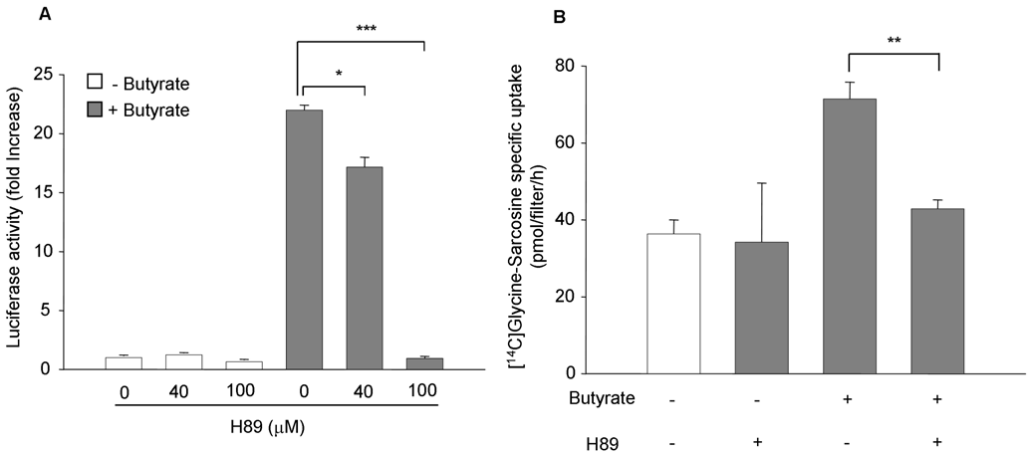
Protein kinase A (PKA) is crucial for butyrate-induced increase of hPepT1 promoter activity in Caco2-BBE cells. A) Caco2-BBE cells transfected with the full-length hPepT1 promoter construct were pre-treated for 1 h with 40 or 100 µM H89, a specific PKA inhibitor, and stimulated with 5 mM butyrate for 24 h. Luciferase activity relative to hPepT1 promoter activity was measured. Data were normalized by Renilla activity and expressed as fold increases compared with control cells. B) Caco2-BBE cells pre-treated or untreated with 100 µM H89 for 1 h were stimulated with 5 mM butyrate for 24 h. hPepT1-mediated [^14^C]Glycine-Sarcosine specific uptake was assessed as described in the Materials and Methods. Values represent means±S.E. of three determinations. *P<0.05; **P<0.005; ***P<0.001.

It was previously shown that protein kinase A (PKA) plays a permissive role in leptin-mediated stimulation of hPepT1 promoter activity [27] as well as in NHE3 promoter activation by butyrate [25]. We therefore investigated if PKA is also involved in butyrate-mediated up-regulation of hPepT1 promoter and transport activities. Cells were pre-incubated for 1 h with H89, a specific PKA inhibitor and then treated or not with butyrate. We found that 100 µM of H89 almost completely inhibited hPepT1 promoter activity (Figure 5A) and significantly reduced ∼85% of hPepT1 transport activity in butyrate-stimulated Caco2-BBE cells (Figure 5B). In contrast, H89 did not affect the basal levels of hPepT1 promoter and transport activities (Figures 5A and B).

Together, these results show that butyrate enhanced hPepT1-mediated transport events and intracellular signaling pathways like PKA may be involved.

### Butyrate treatment does not modify histone H4 acetylation

**Figure 6:**
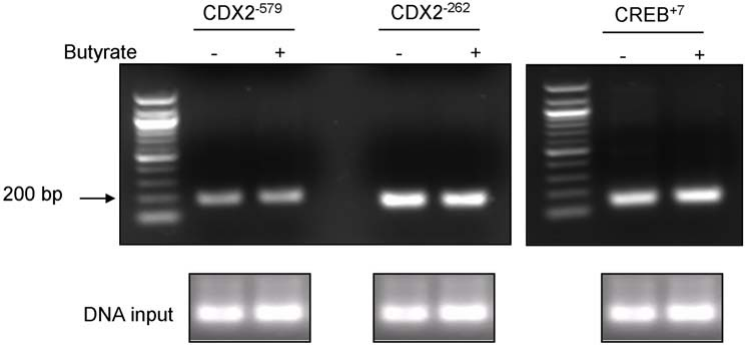
Butyrate does not modify histone H4 acetylation in hPepT1 promoter. Soluble chromatin was prepared from Caco2-BBE cells treated with 5 mM butyrate (+) or vehicle (−) for 24 h. Protein-bound DNA complexes were immunoprecipitated with antibodies against acetyl-histone H4. After cross-link reversal, the purified DNA was amplified with primers specific for Cdx2^−579^, Cdx2^−262^ and CREB^+7^ binding sites.

Butyrate is a potent histone deacetylase inhibitor and its suppression of histone deacetylation has been shown to lead to accumulation of multiacetylated forms of histone especially histone H4 [28]. Histone acetylation alters the compaction of chromatin, preventing DNA high order folding and modifying gene expression. In order to investigate if the acetylation of histone H4 affects hPepT1 promoter activity, we performed a chromatin immunoprecipitation analysis (ChiP). Protein-DNA complexes from butyrate-stimulated or un-stimulated Caco2-BBE cells were cross-linked and immunoprecipitated with anti-acetyl histone H4 antibody. The precipitates were then PCR-amplified with primers specific to each of the Cdx2 binding sites (−579, −262) and the CREB binding site (+7). As shown in Figure 6, butyrate did not induce any significant modification of histone H4 acetylation at hPepT1 promoter regions screened.

### Transcription factors Cdx2 and CREB are crucial for butyrate-induced hPepT1 promoter activity

**Figure 7:**
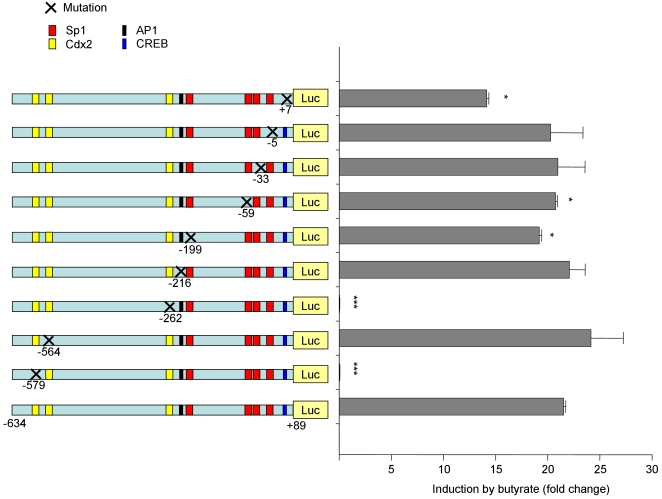
Transcription factors Cdx2 and CREB are crucial for butyrate-induced hPepT1 promoter activity in Caco2-BBE cells. Caco2-BBE cells were transfected with different constructs of hPepT1 promoter that are point mutated at CREB, Sp1, Cdx2, or AP1 sites. Cells were then treated with 5 mM butyrate for 24 h and luciferase activity relative to hPepT1 promoter activity was measured. Data were normalized by Renilla activity and expressed as fold increase in response to butyrate. Values represent means±S.E. of three determinations. *P<0.05; ***P<0.001.

Since it is known that PKA activates the transcription factor CREB [29] and we found here that PKA is involved in the stimulation of hPepT1 promoter activity by butyrate (Figure 5A), the role of CREB in butyrate-mediated activation of hPepT1 promoter was examined. Figure 7 showed that mutation at CREB^+7^ binding site reduced butyrate-induced activation of hPepT1 promoter by ∼30%, confirming the involvement of PKA. We have previously shown that the transcription factor Cdx2 plays an important role in leptin-mediated hPepT1 promoter activation [27] and Cdx2 is known to be activated by butyrate [30]. Here, we tested whether Cdx2 binding is required for butyrate-stimulated hPepT1 promoter activity. Site-directed mutation of individual Cdx2 binding sites were constructed and transfected into Caco2-BBE cells. As shown in Figure 7, mutations of Cdx2 binding sites located at −579 or −262 prevented butyrate-induced increase of hPepT1 promoter activity, whereas mutation at −564 had no effect. To confirm the importance of Cdx2 sites located at −579 or −262, experiment was repeated using 3 different clones and the same results were obtained. It is known that the transcription factor Sp1 has important roles in butyrate-induced activation of several genes [26] and may be also involved in hPepT1 regulation [31], [32]. hPepT1 promoter contains four Sp1 binding sites located at −5, −33, −59 and −199. Point mutations of the Sp1 binding sites at −59 and −199 significantly reduced butyrate-induced increase of hPepT1 promoter activity by ∼10% and 5%, respectively, whereas mutations at −5 and −33 did not affect the stimulation of hPepT1 promoter activity by butyrate (Figure 7). Butyrate was previously implicated in the activation of the transcription factor AP1 in human colon cancer cells [33]. We then investigated the role of AP1 in hPepT1 promoter activation by butyrate. As shown in Figure 7, point mutation at the AP1^−216^ binding site did not affect hPepT1 promoter activation by butyrate.

Together, these results demonstrate that the transcription factors Cdx2 and CREB have crucial roles in the activation of hPepT1 promoter by butyrate. Sp1 might also be involved, albeit to a lesser extent.

### Butyrate increases binding of Cdx2 to hPepT1 promoter

**Figure 8:**
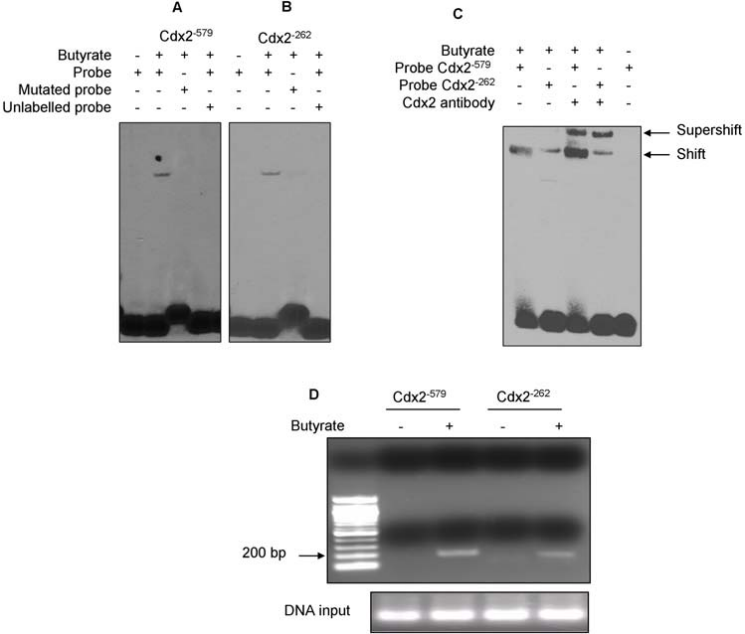
Butyrate induces Cdx2 binding to hPepT1 promoter in Caco2-BBE cells. A, B, C) Caco2-BBE cells were stimulated or not with 5 mM butyrate for 24 h and EMSA were performed using A) Cdx2^−579^ or B) Cdx2^−262^ specific probe. Specificity of complexes was assessed using mutated probe or a 200-fold excess of unlabelled probe. C) Supershift experiments using Cdx2 antibody. D) Soluble chromatin was prepared from Caco2-BBE cells treated with 5 mM butyrate (+) or vehicle (−) for 24 h. Protein-bound DNA complexes were immunoprecipitated with antibodies against Cdx2. After cross-link reversal, the purified DNA was amplified with primers specific for Cdx2^−579^ and Cdx2^−262^ binding sites.

To further confirm the importance of Cdx2 in the activation of hPepT1 promoter, we used electrophoretic mobility shift assays (EMSA) to characterize their binding to hPepT1 promoter at Cdx2^−579^ (Figure 8A) and Cdx2^−262^ (Figure 8B) binding sites. EMSA were carried out using untreated or butyrate-stimulated Caco2-BBE cell extracts, together with biotin-labeled double-stranded oligonucleotides containing the respective consensus Cdx2 binding sites present in hPepT1 promoter or point-mutated binding sites. Butyrate increased the binding of Cdx2 transcription factors to hPepT1 promoter (Figure 8A and B, Lane 2 *vs* Lane 1). However, the retardation complexes were not detected in the presence of labeled oligonucleotides containing point mutations at Cdx2^−579^ and Cdx2 ^−262^ binding sites (Lane 3) or of a 200-fold molar excess of an unlabeled oligonucleotide competitor (Lane 4), indicating that the DNA binding of Cdx2 was sequence-specific. Furthermore, we performed Cdx2 supershift to confirm the binding of Cdx2 to hPepT1 promoter. As shown in Figure 8C, the DNA-protein binding complexes were indeed shifted in the presence of Cdx2 antibody, indicating that Cdx2 bound to hPepT1 promoter.

Collectively, these results confirm the binding of Cdx2 to hPepT1 promoter upon butyrate stimulation.

### In vivo analysis of the putative Cdx2^−579^ and Cdx2^−262^ binding sites in untreated and butyrate-treated Caco2-BBE cells

Since Cdx2 plays a critical role in butyrate-induced hPepT1 expression, we performed a chromatin immunoprecipitation analysis (ChiP) to confirm that Cdx2 binds to hPepT1 promoter. Protein-DNA complexes from butyrate-stimulated or un-stimulated Caco2-BBE cells were cross-linked and immunoprecipitated with anti-Cdx2 antibodies. The precipitates were then PCR-amplified with primers specific to each of the Cdx2 binding sites (−579, −262). Our results showed that Cdx2 binds to its putative sites (−579, −262) in butyrate-stimulated cells but not in un-stimulated cells (Figure 8D).

These results indicate that Cdx2 may have an *in vivo* role in butyrate induction of hPepT1 promoter activity.

### The transcription factor Cdx2 regulates hPepT1 protein expression in Caco2-BBE cells

**Figure 9:**
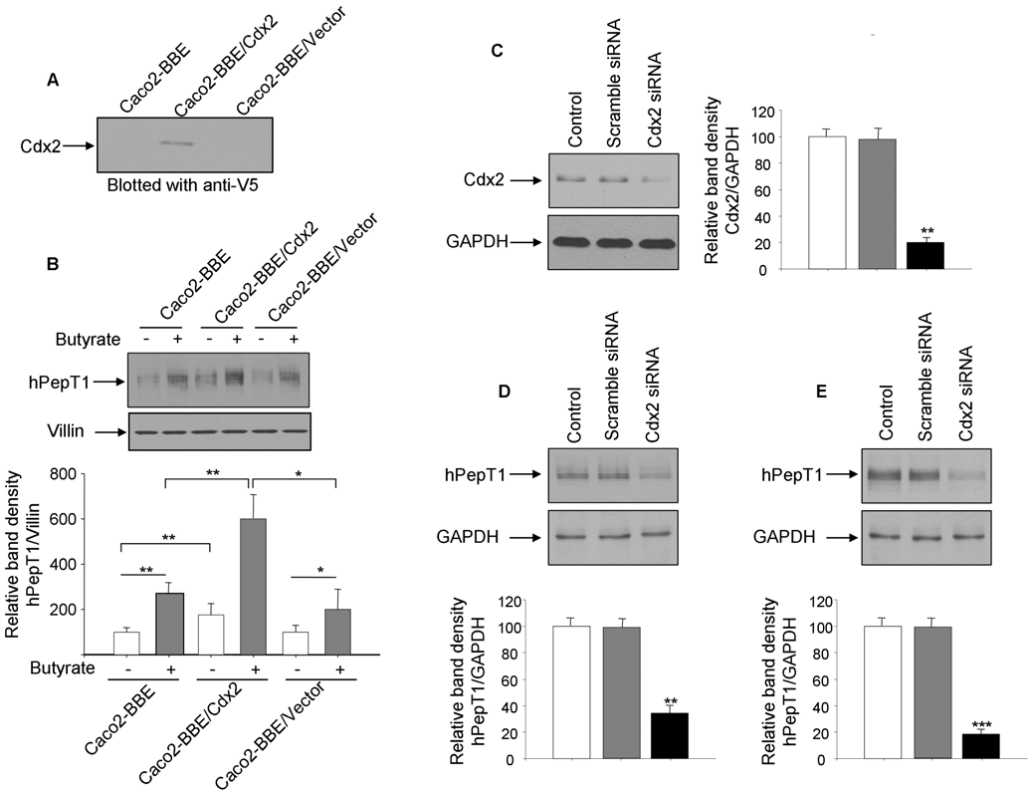
The transcription factor Cdx2 regulates hPepT1 protein expression in Caco2-BBE cells. A) Lysates from wild-type Caco2-BBE cells, cells overexpressing V5-tagged Cdx2 (Caco2-BBE/Cdx2), or cells transfected with vector alone (Caco2-BBE/Vector) were analyzed by SDS-PAGE and immunoblot using anti-V5 antibody. B) Wild-type Caco2-BBE cells, Caco2-BBE/Cdx2 or Caco2-BBE/Vector were treated with 5 mM butyrate (+) or vehicle (−) for 24 h. Membrane proteins were extracted and hPepT1 expression was assessed by immunoblot. C, D) Caco2-BBE cells were transfected for 48 h with Cdx2 siRNA and Cdx2 (C) or hPepT1 (D) expression was assessed by immunoblot. E) 24 h after Cdx2 siRNA transfection, Caco2-BBE cells were treated with 5 mM butyrate for 24 h and hPepT1 expression was assessed by immunoblot. Bar graphs represent blot densitometric quantification. Values represent means±S.E. of four blots from independent experiments. **P<0.005; ***P<0.001.

To confirm the importance of Cdx2 in hPepT1 regulation, we generated a Caco2-BBE cell line over-expressing V5-tagged Cdx2 (Caco2-BBE/Cdx2) and investigated hPepT1 protein expression in this cell line. Immunoblot analysis using an anti-V5 antibody showed a ∼42 kDa band representing Cdx2 in Caco2-BBE/Cdx2, indicating that these cells over-express this transcription factor (Figure 9A). Under the resting state as well as upon 5 mM butyrate treatment, Caco2-BBE/Cdx2 have higher hPepT1 protein expression levels than wild-type cells (Caco2-BBE) or cells transfected with the empty vector (Caco2-BBE/Vector) (Figure 9B). Caco2-BBE cells were then transiently transfected with a Cdx2 siRNA (Figure 9C). We found a decrease of hPepT1 expression in cells transfected with Cdx2 siRNA but not in cells transfected with scramble RNA (Figure 9D, E). This was observed under both resting state (Figure 9D) and butyrate stimulation (Figure 9E).

Together, these results indicate that Cdx2 is important to hPepT1 expression under butyrate stimulation as well as at basal level.

### Butyrate increases hPepT1-mediated inflammation in Caco2-BBE cells

**Figure 10:**
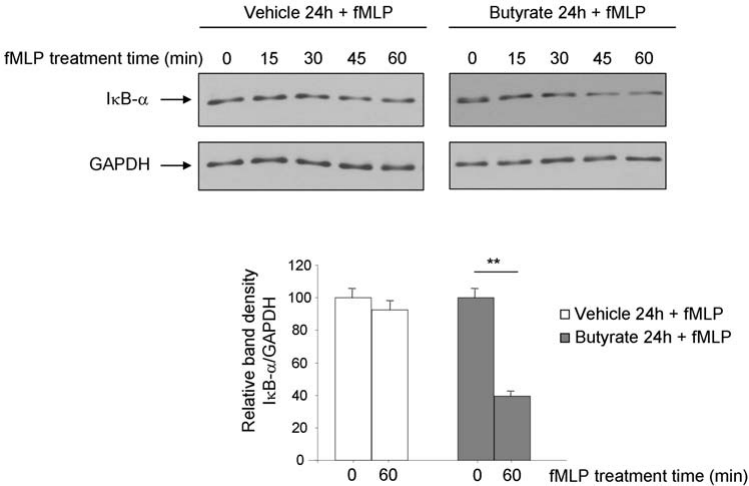
Butyrate increases hPepT1-mediated inflammation in Caco2-BBE cells. Caco2-BBE cells were treated or not (vehicle) with 5 mM butyrate for 24 h. Cells were then washed, incubated in serum-free medium overnight and stimulated with 1 µM fMLP for the indicated times. Cell lysates were analyzed by Western blot using IκB-α antibody. Bar graphs represent the densitometric quantification of IκB-α blots. Values represent means±S.E. of four blots from independent experiments. **P<0.005.

We previously showed that hPepT1 transports pro-inflammatory bacterial peptides such as fMLP (*N*-formyl-methionyl-leucyl-phenylalanine) [34], [35]. As we found that butyrate increased hPepT1 expression and transport activity, we next address if butyrate may play a role in the increased of hPepT1-mediated inflammation. Cells pre-treated or not with 5 mM butyrate were incubated with 1 µM fMLP and degradation of IκB-α was assessed by Western blot. Figure 10 showed a stronger and faster IκB-α degradation in butyrate-treated cells compared to non-treated cells, suggesting the effect of butyrate on hPepT1-mediated inflammation.

### Butyrate increases PepT1 expression and transport activity in mouse colon

**Figure 11:**
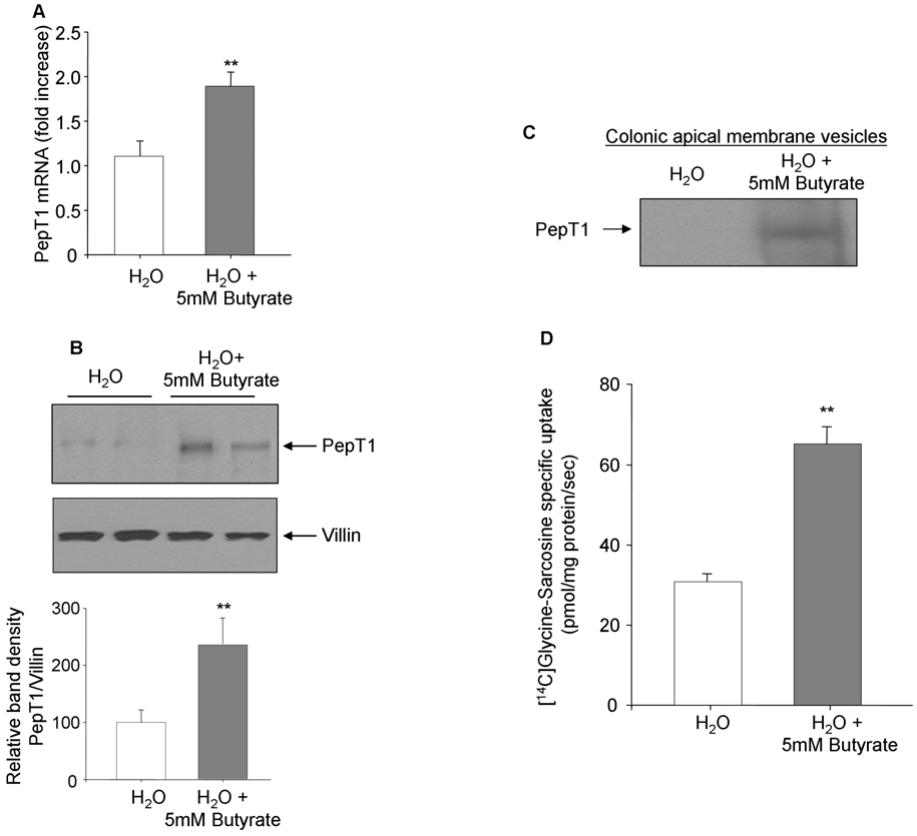
Butyrate increases PepT1 expression and transport activity *in vivo.* C57BL/6 mice were given regular water (H_2_O) or water containing 5 mM butyrate (H_2_O+Butyrate) for 24 h. Mice were then sacrificed and colons were removed. A) PepT1 mRNA level was assessed by real-time RT-PCR (n = 10/condition). B) PepT1 expression in colonic mucosa was analyzed by immunoblot (n = 10/condition) and representative PepT1 blots from different mice are shown. Expression of villin was shown as loading control. Bar graphs represent densitometric quantification of PepT1 bands. Values represent means±S.E. of four blots from independent experiments. C) Colonic apical membrane vesicles were prepared from H_2_O− or H_2_O+butyrate-treated mice (n = 25/condition) and analyzed for PepT1 expression by Western blot. D) PepT1-mediated specific uptake of [^14^C]Glycine-Sarcosine was measured in the colonic apical membrane vesicles (n = 25/condition). Values represent means±S.E. of three determinations. **P<0.005.

To investigate the *in vivo* effect of butyrate on PepT1 expression, mice were treated for 24 h with 5 mM butyrate added to the drinking water and PepT1 expression level in the colonic mucosa was examined. It has previously shown that the presence of butyrate in the drinking water did not significantly modify the volume of water ingested by animals [36], [37]. Using real-time RT-PCR, we found that butyrate treatment induced a significant increase of PepT1 mRNA expression by ∼2-fold compared with untreated mice (Figure 11A). To confirm this result, PepT1 protein expression level was assessed by Western-blot. PepT1 protein was barely detected in scrapped colonic mucosa from untreated mice but clearly detected in colonic mucosa from mice treated with butyrate (Figure 11B). We next examined the effect of butyrate on PepT1 expression and transport activity in mouse colonic apical membrane vesicles. The membrane vesicles were prepared as described in Materials and Methods and analysed by Western blot for PepT1 expression. We found that butyrate significantly increased PepT1 expression (Figure 11C). In agreement, the specific PepT1-mediated transport of [^14^C]Glycine-Sarcosine in colonic apical membrane vesicles from butyrate-treated mice was significantly increased by ∼2 times compared with untreated mice (Figure 11D). These results indicate that butyrate enhances PepT1 expression and function in mouse colon. These confirm our *in vitro* findings showing that butyrate is a potent PepT1 expression inducer.

## Discussion

The human di/tripeptide transporter hPepT1 is not expressed [22] or barely detected [23] in normal colon. However, its expression can be induced in inflamed colon as we have previously shown [22]. Other studies also showed the expression of hPepT1 in colonic epithelial cells from patients with short gut syndrome [23]. These observations indicate that inflammatory signaling pathways may regulate the expression of colonic hPepT1.

Here we showed that treatment of human intestinal epithelial Caco2-BBE cells for 24 h with 5 mM butyrate, an end product in the breakdown of carbohydrates by anaerobic bacteria, increased mRNA and protein expressions as well as the activity of this transporter. However, in normal colon, hPepT1 expression is not detected although butyrate concentration in the lumen is 10–15 mM [1], [3]. The expression of hPepT1 induced in inflammation could be explained by a dysfunction of butyrate metabolism in colonocytes. Indeed, butyrate oxidation in colonocytes from IBD patients is significantly decreased [38], [39]. A decrease of butyrate oxidation up to 80% was also found in DSS-induced colitis in mice [40]. This oxidation default can induce an intracellular accumulation of butyrate in colonocytes, which may be responsible for PepT1 expression. The monocarboxylic transporter (MCT)-1 expressed at the apical membrane of colonic epithelial cells may also play a major role in butyrate uptake [6]. Therefore, MCT-1 expression level limits the intracellular accumulation of butyrate [41]. This has previously been confirmed by suppressing the expression of MCT-1 *in vitro*, resulting in a marked inhibition of the ability of butyrate to regulate the expression of several target genes [41]. It is possible that MCT-1 expression/activity varies between inflamed and non-inflamed colonocytes, differentially regulating colonic intracellular concentrations of butyrate, thereby modulating gene expression.

In the present study, we demonstrated that the transcription factors Cdx2 and CREB are crucial for the butyrate-induced increase of hPepT1 expression in Caco2-BBE cells. In adult mammals, the expression of Cdx2 is largely restricted to the epithelia throughout the small and large intestines [42]. Cdx2 appears to play critical roles in gut differentiation, proliferation and neoplasia [43], and it was recently demonstrated that the gastric mucosa of transgenic Cdx2-overexpressing mice are morphologically changed to intestinal metaplastic mucosa including microvilli and PepT1 expression [43], [44]. In agreement with our finding, it has recently been demonstrated that the intestine-specific transcription factor Cdx2 is stimulated by butyrate [30]. hPepT1 promoter contains three potential Cdx2 binding sites [27] and here we showed that two of these located at −579 and −262 are crucial for the activation of hPepT1 promoter by butyrate. These results are in agreement with previous studies that have reported the crucial role of Cdx2 in hPepT1 basal or leptin-stimulated activity [27], [31]. We have also demonstrated the importance of the transcription factor CREB in butyrate-mediated up-regulation of hPepT1 expression. Several studies have shown the importance of CREB in butyrate-induced gene expression. For example, short-chain fatty acids regulate tyrosine hydroxylase gene expression through a cAMP-dependent signaling pathway [45]. In another report, additional evidence has been provided to show that exposure to butyrate rapidly activates the MAP kinase pathway, which is associated with increased CREB phosphorylation [46]. The phosphorylation of CREB is likely to play a role in hPepT1 expression since we found that the PKA inhibitor H89 abolished butyrate-mediated increase of hPepT1 transport activity. However, knocking-out CREB binding site results in a ∼30% decrease of promoter activity. This indicates that PKA can regulate transcription factors involved in hPepT1 regulation other than CREB. PKA has been also found to be important for butyrate-mediated regulation of NHE3 expression [25]. This indicates that butyrate stimulates a PKA-dependent functional transcriptional regulation of hPepT1 and NHE3 in epithelial cells. However, it seems that Cdx2 does not play a role in the regulation NHE3 or the human γ-epithelial sodium channel expression [24], [25], [26] suggesting the specific role of Cdx2 in regulating hPepT1 expression/function.

It has been known that butyrate induces an accumulation of multiacetylated forms of histones [28]. Histone acetylation alters the chromatin structure at the nucleosomal level, facilitating changes in DNA transcription. In this study, we were not able to detect any change in histone H4 acetylation. However we cannot exclude the possibility that other histones may be hyperacetylated or histone H4 may be acetylated upstream or downstream of the areas tested.

Our data demonstrated that butyrate, both *in vivo* and *in vitro*, induced PepT1 expression/activity. It has been reported that PepT1 transports pro-inflammatory bacterial peptides, such as muramyl dipeptide (MDP) [47] or *N*-formyl-methionyl-leucyl-phenylalanine (fMLP) [20], [34], [35], which participate in intestinal inflammation. Butyrate has been shown to exert anti-inflammatory effects in several cell types as well as intestinal biopsy specimens [48], [49]. Here we suggest that intracellular accumulation of butyrate in colonocytes may be involved in intestinal inflammation via hPepT1 expression and pro-inflammatory peptide transport. This speculation is supported by i) our results showing that intestinal epithelial cells pre-treated with butyrate were more sensitive than un-treated cells to fMLP induces IκB-α degradation and by ii) publications demonstrating that *in vivo* perfusion of fMLP induces PepT1-mediated colonic inflammation in a short bowel model where PepT1 is expressed in colonic epithelial cells [50], [51]. These findings provide important new insights to understand the regulation of hPepT1 expression in intestinal inflammation.

## Materials and Methods

### Cell culture and transfection

Caco2-BBE cells were grown in DMEM supplemented with 14 mM NaHCO3, 10% FBS, and penicillin/streptomycin (Invitrogen, Grand Island, NY). Caco2-BBE cells stably transfected with Cdx2-pcDNA3 plasmid [27] were maintained in culture medium containing 1.2 mg/ml G418 (Invitrogen). Cells were kept at 37°C in a 5% CO_2_ atmosphere and 90% humidity, and the medium was changed each 2 days. Cells were grown on 6-well plastic plates or on permeable supports (area = 1 cm^2^; pore size, 0.4 µm; Transwell-Clear polyester membranes from Costar VWR, Suwanee, GA).

### Reagents

SCFAs, Actinomycin D and fMLP were obtained from Sigma-Aldrich (St. Louis, MO). H89 was purchased from Biosource (Camarillo, CA).

### Animals

Female C57BL/6 mice (8 weeks, 18–22 g, Jackson Laboratories, Bar Harbor, ME) used for this study were group-housed under a controlled temperature (25°C) and photoperiod (12:12-hours light-dark cycle), and allowed unrestricted access to standard diet and tap water. All animal procedures were approved by the Animal Care Committee of Emory University and were conducted in accordance to the *Guide for the Care of Use of Laboratory Animals* from the US Public Health Service.

### Preparation of the reporter constructs

Analysis with the TFSEARCH prediction program (www.cbrc.jp/) revealed that the cloned promoter region contained multiple putative biding sites for transcription factors. Site directed mutations at the putative CREB^+7^, Cdx2^−579,−564,−262^, AP1^−216^ and Sp1^−199,−59,−33,−5^ binding sites of the −634/+89 hPepT1 construct were introduced using the Quick Change XL Site Directed Mutagenesis Kit (Stratagene, La Jolla, CA) with the following primers: hPepT1 promoter with mutated CREB^+7^, sense 5′-CAA CGG GGC CGG GCC TGG AAT TCA GGT CGG AGG AGT AG-3′ and antisense 5′-CTA CTC CTC CGA CCT GAA TTC CAG GCC CGG CCC CGT TG-3′; hPepT1 promoter with the first Cdx2^−579^ site mutated, sense 5′-GAA ATG TAG AAT CCC CCA GAG ATG CTT TCA AAG GTT-3′ and antisense 5′-AAC CTT TGA AAG CAT CTC TGG GGG ATT CTA CAT TTC-3′; hPepT1 promoter with the second Cdx2^−564^ site mutated, sense 5′-GGT TGA ATC TCA AAA TGA AGC CAC ACA CAC ACT CT-5′ and antisense 5′-AGA GTG TGT GTG TGG CTT CAT TTT GAG ATT CAA CC-3′; hPepT1 promoter with the third Cdx2^−262^ site mutated, sense 5′- AAC CTC CCC TTA GAC TTC TTC GAA ATG CAC ATT CTG G 3′ and antisense 5′-CCA GAA TGT GCA TTT CGA AGA AGT CTA AGG GGA GGT T-3′; hPepT1 promoter with the AP1^−216^ site mutated, sense 5′-AGC CCC GAC CTC CTG AGT CTG CTG GCC GGG GGG TGG-3′ and antisense 5′-CCA CCC CCC GGC CAG CAG ACT CAG GAG GTC GGG GCT-3′; hPepT1 promoter with the first Sp1^−199^ site mutated, sense 5′-CAG CTG GCC GGG GGG TTG GGC CTG GGA ATC CGC GTT-3′ and antisense 5′-AAC GCG GAT TCC CAG GCC CCA CCC CCC GGC CAG CTG-3′; hPepT1 promoter with the second Sp1^−59^ site mutated, sense 5′-CTC TGC TCC CCG CAG CAC CGT CCC CCG GGT GGA GCC-3′ and antisense 5′-GGC TCC ACC CGG GGG ACG GTG CTG CGG GGA GCA GAG-3′; hPepT1 promoter with the third Sp1^−33^ site mutated, sense 5′-TGG AGC CGG CGG CCC CTC CTC GCA GAG CTG GGG CTG-3′ and antisense 5′-CAG CCC CAG CTC TGC GAG GAG GGG CCG CCG GCT CCA-3′; hPepT1 promoter with the fourth Sp1^−5^ site mutated, 5′-GTA CCT GGG GCA ACG GGG ACG GGC CTG GAC GTC AGG-3′ and antisense 5′-CCT GAC GTC CAG GCC CGT CCC CGT TGC CCC AGG TAC-3′. The PCR conditions consisted of 95°C for 30 sec, followed by 12 cycles of 95°C for 30 sec, 55°C for 2 min and 68°C for 10 min. All mutants were confirmed by DNA sequencing.

### siRNA study

Cdx2 siRNA (5′-GCC AUA GAC CUA CAG ACC Utt-3′) was purchased from Ambion (Austin, TX) and transfected into Caco2-BBE cells using the siPORT NeoFX transfection reagent (Ambion). 48 hours after transfection, cells were treated with 5 mM butyrate for 24 h, harvested and analyzed by Western blot.

### Dual-luciferase reporter assay

Caco2-BBE cells were transfected with 5 ng of a construct encoding Renilla luciferase (Promega, Madison, WI) and 2 µg of the relevant hPepT1 promoter construct using 10 µg/ml lipofectin (Invitrogen). After stimulation, the resulting luminescence was measured for 10 s in a luminometer (Luminoskan, Thermal Labsystems, MA). Each luciferase activity was normalized based on the control Renilla luciferase activity. Extracts were analyzed in triplicate, and each experiment was performed for at least three times.

### Generation of polyclonal antibodies to PepT1

Based on a computerized predictive model for antigenicity and uniqueness, we designed two synthetic peptides (H_2_N-FRHRSKAYPKREHWC-COOH) and H_2_N-RLEKSNPYFMSGANSQKQN), corresponding to residues 248–261 and 689–708 of the deduced hPepT1 protein. The peptides were coupled to keyhole limpet haemocyanin, and used to raise two antibodies: (anti-PepT1^248–261^; anti-PepT1^689–708^) in rabbits via a standard 80-day immunization protocol. The reactivity of the resulting antisera against the appropriate peptide was tested by enzyme-linked immunosorbent assay (ELISA), and the antibodies were affinity purified against the synthetic peptide. Anti PepT1^689–708^ is specifically directed to human PepT1 and anti-PepT1 ^248–261^ is directed to mouse and human PepT1 [20], [22].

### Protein extraction and Western blot analysis

Total and membrane proteins were extracted as previously described [27]. Proteins were resolved on 10% polyacrylamide gels and transferred to PVDF membranes (Bio-Rad, Hercules, CA). Membranes were probed overnight at 4°C with anti-villin, anti-GAPDH, anti-IκB-α (Santa Cruz Biotechnology, Inc; Santa Cruz, CA), anti-V5 (Invitrogen), anti-sodium potassium ATPase (Abcam Inc; Cambridge, MA) or the appropriate anti-PepT1. After washing, membranes were further incubated for 1 h at room temperature with appropriate HRP-conjugated secondary antibodies (Amersham Biosciences, Buckinghamshire, England). Immunoreactive proteins were detected with the enhanced chemiluminescence detection system (Amersham Biosciences).

### RNA extraction and RT-PCR

Total RNA was extracted using TRIzol reagent (Invitrogen) and reverse transcribed using Thermoscript™ RTPCR System (Invitrogen). The PCR was carried out in a final volume of 50 µl using 2 µl of RT first-strand cDNA product, 1 µM of each forward and reverse primer, and PCR SuperMix (Invitrogen). The primers used were: hPepT1 sense 5′-CGC CAT GGG AAT GTC CAA ATC ACA CAG T-3′ antisense 5′-CAT CTG TTT CTG TGA ATT GGC CCC-3′; β-actin sense 5′-GTC ACC CAC ACT GTG CCC ATC-3′ antisense 5′-ACG GAG TAC TTG CGC TCA GGA-3′.

### Real-time RT-PCR

Real-time PCR with iCycler sequence detection system (Bio-Rad) was used to assess PepT1 transcripts. Briefly, 50 ng of reverse-transcribed cDNA was amplified followed by 40 cycles of 95°C for 15 s and 60°C for 1 min, using 10 µM of gene-specific primers and the iQ SYBR Green Supermix (Biorad). The GAPDH or 36B4 expression levels were used as housekeeping genes, and fold-induction was calculated using the Ct method as follows: ΔΔCT = (*C*t_Target_−*C*t_housekeeping_)treatment−(*C*t_Target_−*C*t_housekeeping_)nontreatment, and the final data were derived from 2^−ΔΔCT^. The primers used were as follows: hPepT1 sense 5′-CGT GCA CGT AGC ACT GTC CAT-3′ hPepT1 antisense 5′-GGC TTG ATT CCT CCT GTA CCA-3′; hGAPDH sense 5′-GTC GGA GTC AAC GGA TTT GG-3′ hGAPDH antisense 5′-AAG CTT CCC GTT CTC AGC CT-3′; mousePepT1 sense 5′-CGT GCA AGT AGCACTGTC CAT-3′; mousePepT1 antisense 5′-GGC TTG ATT CCT CCT GTA CCA-3′; mouse36B4 sense 5′-TCC AGG CTT TGG GCA TCA-3′ mouse36B4 antisense 5′-CTT TAT CAG CTG CAC ATC ACT CAG A-3′.

### Electrophoretic mobility shift assay (EMSA)

Cdx2 or CREB-DNA binding activities were analyzed in cellular extracts prepared in totex buffer (20 mM HEPES/pH7.9, 350 mM NaCl, 20% glycerol, 1% NP-40, 1 mM MgCl_2_, 0.5 mM EDTA, 0.1 mM EGTA, protease inhibitors). Samples (5 µg) were incubated for 15 min at room temperature with a biotin-labeled double-stranded oligonucleotide (Pierce Rockford, IL) containing Cdx2^−579^, Cdx2^−262^ or CREB^+7^ binding site. Complexes were resolved by electrophoresis on 5% TBE gels in 0.5× TBE buffer. Gels were transferred to Biodyne B Pre-cut Modified Nylon Membranes (Pierce) and complexes were visualized using the Chemiluminescent Nucleic Acid Detection System (Pierce). The specificity of the complexes was analyzed by incubation with a 200-fold excess of unlabeled oligonucleotides as well as a mutated probe. Supershift assay was performed using 2 µg of Cdx2 antibody (Zymed Laboratories Inc. laboratories, San Francisco, CA).

### hPepT1 mRNA stability assay and Northern blot

For mRNA decay experiments, Caco2-BBE cells pretreated with 5 µg/ml actinomycin D (AcD) for 1 h to arrest transcription were incubated with 5 mM butyrate for indicated times. The hPepT1 mRNA was quantified by real-time RT-PCR as described above. Northern blot analysis was performed using the North2South Complete Biotin Random Prime Labeling and Detection Kit (Pierce, Rockford, IL) with 20 µg of template total RNAs and probe generated by RT-PCR with primers as followed: hPepT1sense 5′-GGA GCC CTG GGA GCC GCC GCC ATG-3′ and hPepT1 antisense 5′-TTG TTG CCT GCA GTG TCC ACC TGG-3′.

### Chromatin immunoprecipitation assay

Acetyl-histone H4 and Cdx2 chromatin immunoprecipitation assay (ChIP) was performed using the ChIP assay kit (Upstate Cell Signaling Solutions, Lake Placid, NY). Briefly, after stimulation cells were fixed with 1% formaldehyde for 10 min at 37°C (protein to DNA cross-linking), resuspended in SDS lysis buffer for 10 min on ice. Cells were then sonicated to shear the DNA into 200–1000 bp fragments and centrifuged. The supernatant was diluted in ChIP dilution buffer and pre-cleared with protein A-agarose/salmon sperm DNA to reduce the nonspecific background. The samples were then immunoprecipitated with anti-acetyl-histone H4 or anti-Cdx2 antibodies overnight at 4°C. The complexes were collected in protein A-agarose/salmon sperm DNA slurry 1 h a 4°C, and then washed once each with the provided low salt, high salt, and LiCl wash buffers, and twice in TE buffer (10 mM Tris-HCl, pH 8, 1 mM EDTA). The immunoprecipitated chromatin was eluted from the protein A using freshly prepared elution buffer (10 mM NaHCO_3_, 1% SDS) and the protein-DNA cross-links were reversed by treatment with 200 mM NaCl at 65°C for 4 h. The DNA was purified by incubation with proteinase K at 45°C for 1 h, followed by phenol/chloroform extraction and ethanol precipitation. The Cdx2 promoter elements in the immunoprecipitates were detected by PCR using specific primers (Cdx^−579^ sense 5′-TCT TAA AGA AAG GAA ATG TAG AAT CC-3′ Cdx^−579^ antisense 5′-TGT GTG TGT GAA TGA GGA TTG A-3′; Cdx^−262^ sense 5′-CCC ACA GTG GTT TCC AAA GT-3′ Cdx^−262^ antisense 5′-AGC CAG TCT AAA CGC GGA TT-3′). The CREB^+7^ promoter elements were detected using specific primers: CREB^+7^ sense 5′-CTC GGG AGC ACG GAC CTC T-3′ CREB^+7^ antisense 5′-CCT GGC AGG TGC TAC TCC TC-3′.

### Uptake experiments

Caco2-BBE cells grown on filters for 15 days were treated with butyrate, washed and incubated with HBSS^+^-10 mM HEPES (pH 7.4) in the basolateral compartment and with HBSS^+^-10 mM MES (pH 6.2) in the apical compartment for 15 min at 37°C. Apical and basolateral compartments were then incubated for 15 min at room temperature with HBSS^+^-10 mM MES (pH 6.2) containing 20 µM [^14^C]Gly-Sar±20 mM Gly-Leu and HBSS^+^-10 mM MES (pH 7.2), respectively. Cells were washed twice in ice-cold PBS, filters were cut, and cell-associated radioactivity was determined using a β-counter. The results, expressed as specific uptake of [^14^C]Gly-Sar mediated by hPepT1, are calculated as follows: (Uptake of [^14^C]Gly-Sar)−(Uptake of [^14^C]Gly-Sar + Gly-Leu).

The kinetic parameters of peptide transport in Caco2-BBE monolayers were examined using different doses of [^3^H]KPV and the hPepT1-mediated specific uptakes of [^3^H]KPV were performed as described above.

### Preparation of mouse colonic apical membrane vesicles and in vivo uptake experiment

Colonic apical membranes were prepared from mucosal scraping (n = 25 mice/condition) as previously described [52], [53]. Briefly, the mucosa was homogenized in a buffer containing 60 mM mannitol, 12 mM Tris-HCl pH 7.4, 10 mM EGTA, and protease inhibitors. The homogenate was centrifuged at 3,000 g for 15 min (Step1). The supernatant was incubated with ice-cold 10 mM MgCl_2_ for 15 min and centrifuged at 27,000 g for 30 min (Step2). The pellet was resuspended in the homogenization buffer. Steps 1 and 2 were repeated. The pellet was homogenized in ice-cold preloading buffer (100 mM KCl, 100 mM mannitol, 20 mM HEPES/Tris-HCl pH 7.4, protease inhibitors) and centrifuged at 27,000 g for 30 min. The purified colonic apical membrane vesicles were resuspended in the preloading buffer. *In vivo* uptake experiments were performed using a rapid filtration technique with Millipore filters (HAWP type, 0.45 µm). Uptake of [^14^C]Gly-Sar was performed for 10 s at room temperature using transport buffer (HBSS^+^-10 mM MES pH 6.2, 100 mM mannitol) containing 300 µg of colonic apical membranes and 20 µM [^14^C]Gly-Sar±20 mM Gly-Leu, following by addition of ice-cold stop solution (2 mM HEPES/Tris pH 7.4, 210 mM KCl) and filtration. The filters were then washed twice with stop solution and the radioactivity was determined using a β-counter.

### Statistical analysis

All evaluations were performed using SigmaPlot (SPSS, Chicago, IL) and InStat v3.06 (GraphPad, San Diego, CA) softwares, with data reported as means±S.E. Multiple groups were compared by ANOVA, using Tukey's post-hoc test. *P*<0.05 was considered significant.
